# Isolation of acephate-degrading bacteria and phytoremediation–microbial remediation from soil for the project of water diversion from the Yangtze River to Chaohu Lake

**DOI:** 10.3389/fmicb.2025.1675842

**Published:** 2025-10-24

**Authors:** Huili Wang, Jielun Chang, Chang Pan, Dongsheng Jiang, Yemei Wang, Qin Yin, Xi Chen, Xi Liao, Manman Li, Xiaoke Zhang

**Affiliations:** Engineering Technology Research Center for Aquatic Organism Conservation and Water Ecosystem Restoration in University of Anhui Province, College of Life Science, Anqing Normal University, Anqing, China

**Keywords:** acephate, riparian zones, bioremediation, pesticide degradation, soil contamination

## Abstract

**Introduction:**

Efficient and safe governance of soil contaminated with organophosphate pesticides is of crucial significance for the protection of the ecosystem. This study focuses on soils from typical riparian zones along the project of water diversion from the Yangtze River to Chaohu Lake, aiming to screen acephate-degrading microorganisms and to systematically evaluate their degradation efficiency.

**Methods:**

Acephate-degrading bacteria were isolated from soil via enrichment culture with acephate as the sole carbon source, and their degradation efficiency was subsequently evaluated. Subsequently, a pot experiment was designed to investigate the efficiency of the combined remediation of soil acephate through the synergistic action of the isolated bacteria and plants.

**Results:**

Five acephate-degrading strains were isolated and identified via 16S rDNA sequencing as *Enterobacter cloacae*, *Enterobacter hormaechei*, *Bacillus badius*, *Sphingobacterium spiritivorum*, and *Serratia nematodiphila*. Although all strains degraded acephate, their efficiencies differed significantly. Except for the 50 mg L^−1^ acephate condition with added glucose, *B. badius* consistently exhibited higher degradation efficiency across all tested conditions. Furthermore, increasing acephate concentration in the culture medium from 10 to 50 mg L^−1^ reduced degradation efficiency across strains. However, adding 0.1 g L^−1^ glucose enhanced degradation rates for all strains, with *B. badius* achieving the highest degradation efficiency (76.17% at 10 mg L^−1^ acephate). For combined experiments, we paired *B. badius* (with superior *in vitro* degradation performance) with *Persicaria hydropiper*, and *S. spiritivorum* with *Carex dimorpholepis*. At both 200 μg kg^−1^ and 1,000 μg kg^−1^ soil acephate concentrations, combined remediation efficiencies exceeded those of microbes or plants alone. The combination of *B. badius* and *P. hydropiper* achieved the highest removal rate of 91.27% at the 1,000 μg kg^−1^ acephate concentration.

**Conclusion:**

These findings significantly enrich the repository of acephate-degrading bacteria and demonstrate that combined remediation with *B. badius* and *P. hydropiper* is an effective strategy for the bioremediation of acephate-contaminated soils within the project of water diversion from the Yangtze River to Chaohu Lake.

## 1 Introduction

Acephate has been widely applied in the management of pests across vegetable, fruit, and other crop species, attributable to its high insecticidal efficacy and relatively low toxicity (Hou, 2018). Following the comprehensive prohibition of the highly toxic pesticide methamidophos in China, acephate became the predominant alternative, exhibiting consistently increasing annual usage (Hua, 2014). However, the utilization efficiency of acephate remains low, with only approximately 0.1% of the active ingredient reaching the target organisms, whereas up to 99.9% disperses into ecological ecosystems. For instance, Raj and Krishnan (2023) identified acephate in wastewater, and Omwenga et al. (2021) documented concerning residue levels in common vegetable cultivars. Yan et al. (2024) evaluated the potential risks of acephate to bees and earthworms and found that acephate products had a significant impact on the body weight of earthworms. Dhanushka and Peiris (2017) found that long-term exposure to acephate can lead to alterations in human sperm structure and function, and reduce semen quality, posing potential risks to both the ecological environment and human health (Xie et al., 2008; Tao, 2022). Therefore, effective remediation of acephate-contaminated environments is critical for protecting ecological security and public health.

Currently, remediation strategies for organophosphorus pesticides primarily involve physical, chemical, and biological degradation approaches. Conventional physicochemical methods, though widely employed, frequently result in incomplete degradation, leading to the transformation of soil pesticide residues into secondary pollutants that threaten ecological safety (Mohamed et al., 1998; Tu, 1993). In contrast, biodegradation—particularly microbial degradation—demonstrates greater potential in efficiency and environmental compatibility. Studies indicate microbial degradation achieves removal rates exceeding 90%, with efficiencies over tenfold higher than those of physical or chemical treatments (Giri et al., 2021; Kumar et al., 2018). In natural environments, microbial metabolic activity predominantly mediates the degradation of most organophosphorus pesticides (Sun, 2007). Consequently, applying microorganisms with high degradation capacities has become a focal strategy for remediating soils contaminated with organophosphorus pesticides, especially acephate. For example, Ren et al. (2020) reported that *Bacillus paramycoides* degraded 500 mg L^−1^ acephate at a rate of 76% within 48 h. Wang et al. (2013) demonstrated that the bacterial strain *Hyphomicrobium* sp. achieved complete degradation of 100 mg kg^−1^ acephate within 9 days at 30 °C and pH 6.8. Similarly, Yu et al. (2010) isolated *Stenotrophomonas* sp. and *Pseudomonas* sp. from pesticide-contaminated soils; under conditions of 500–1,000 mg L^−1^ acephate, 30 °C, pH 8, and 2.5% inoculum, these strains mineralized acephate into phosphate within 1 week, achieving degradation rates approaching 80%.

The principal mechanism for microbial degradation of organophosphorus pesticides entails enzymatic reactions, wherein cleavage of P–O, P–S, and P–N bonds mediates breakdown (Wei et al., 2022). For instance, Lourthuraj et al. (2022) confirmed that *E. aerogenes* and *S. pyogenes* secrete organophosphorus hydrolase extracellularly, enhancing chlorpyrifos degradation. However, microbial degradation exhibits high sensitivity to environmental conditions and is influenced by factors including indigenous microorganism abundance and functionality, alongside soil plant community composition (Bu et al., 2023). Previous studies indicate that laboratory-screened strains frequently underperform in field applications, attributable to divergent soil physicochemical properties, competition with native microbes for ecological niches, and insufficient environmental adaptability (Sun et al., 2019). Plant–microbe combined remediation represents a promising strategy, as plant root exudates provide energy and nutrients that enhance microbial activity and degradation efficiency (Oram et al., 2025). For instance, Ma isolated two bacterial strains (*Enterobacter* sp. and *Achromobacter* sp.) and a fungal strain (*Scedosporium* sp.), applying them with *Lolium multiflorum* to remediate carbendazim-contaminated soil. After 21 days, carbendazim removal efficiencies reached 57.66–78.90%, significantly exceeding microbial-only remediation (41.77–62.9%) (Ma, 2022). Similarly, Chang et al. (2025) confirmed that the combination of *Acinetobacter seifertii* and *Carex dimorpholepis* achieved a 93.27% degradation rate for Ethoprophos within 30 days, and this degradation efficiency significantly outperformed that of individual microbial or plant treatments. Lin and You (2009) demonstrated that the combination of *Arthrobacter* sp. and the plants *Sorghum drummondii*, *Medicago sativa*, and *Lolium perenne* significantly enhanced chlorpyrifos degradation in contaminated soil, surpassing those of individual plants. Consequently, plant–microbe combined remediation demonstrates substantial potential for acephate-contaminated soil remediation, although relevant studies remain limited.

As a strategic water resource allocation project in Anhui Province, the project of water diversion from the Yangtze River to Chaohu Lake (YC-project) conveys water through Caizi Lake, Kongcheng River, Luobu River, and Baishi River before discharging into Chaohu Lake. Recent studies report acephate accumulation in both water bodies and sediments along the YC-project route, exhibiting variable enrichment levels with moderate environmental risk (Song et al., 2022). Literature documents numerous high-efficiency acephate-degrading microorganisms, including *Acinetobacter* sp., *Pseudomonas* sp., *Exiguobacterium* sp., and *Rhodococcus* sp. (Phugare et al., 2012; Pinjari et al., 2012), predominantly isolated from pesticide manufacturing sites or pesticide-contaminated soils. Nevertheless, riparian zones constitute ecotones between terrestrial and aquatic systems that differ fundamentally from agroecosystems, raising unresolved questions regarding the presence of efficient acephate-degrading strains in these transitional environments.

To address tacephate contamination in soils along the YC-project, this study sampled rhizosphere soils from riparian zones, isolated acephate-degrading strains, and conducted pot experiments to evaluate the efficacy of combined plant–microbe remediation. We hypothesize that combining acephate-degrading bacteria with plant-assisted remediation can enhance pesticide removal from soil. Our findings will enrich the repository of acephate-degrading microbial resources and establish a scientific foundation for the remediation of contaminated soils along the project’s route.

## 2 Materials and methods

### 2.1 Soil sample collection

**Figure 1:**
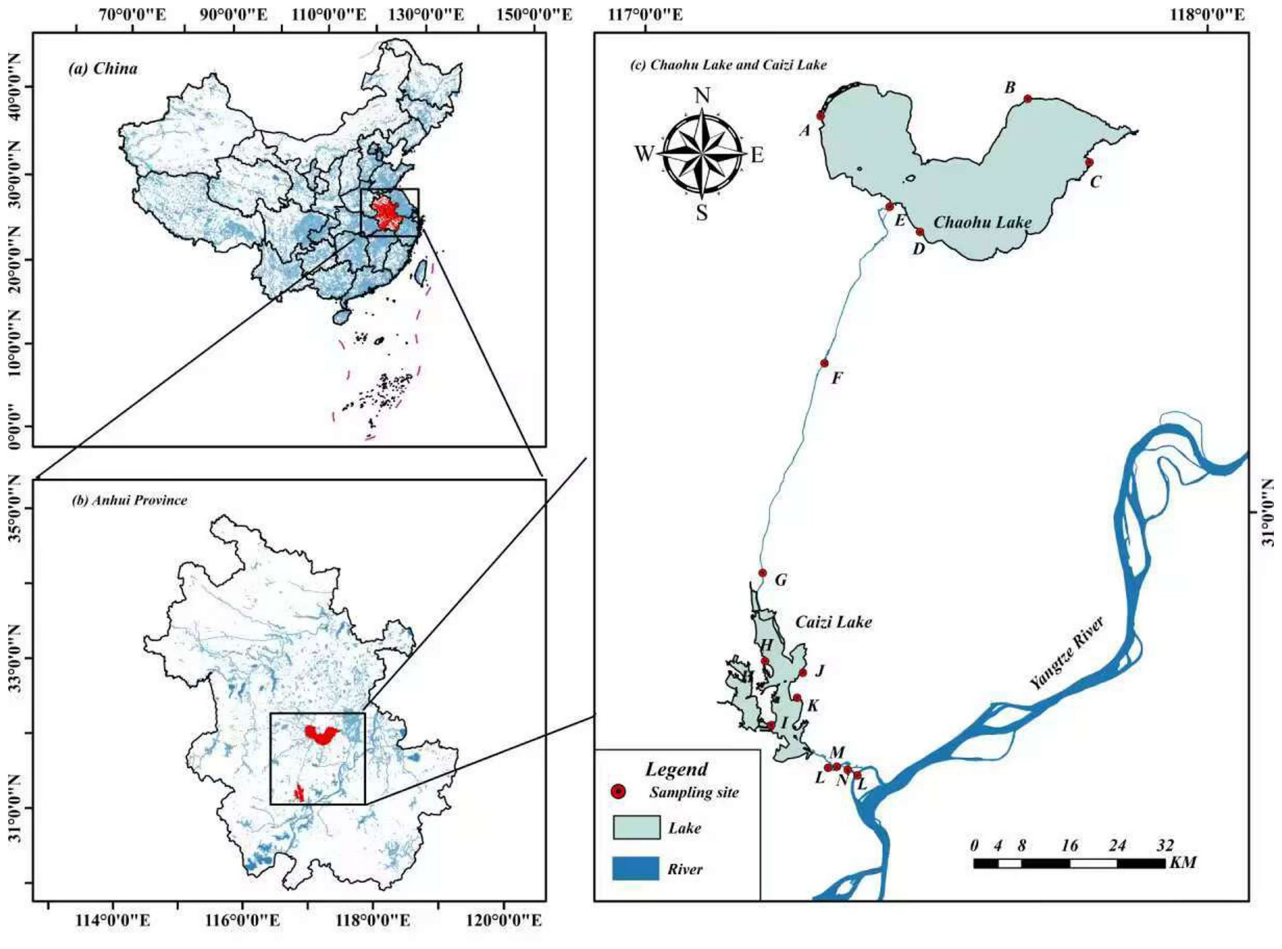
Distribution of sampling sites.

In October 2022, 15 sampling sites were systematically established along the riparian zones of the YC-project (Figure 1). These sites were selected to represent regional typical vegetation types and were evenly distributed along the YC-project route. At each location, 3–6 quadrats (1 m × 1 m) were positioned perpendicular to the riverbank or lakeshore. Rhizosphere soil samples were collected from densely vegetation areas using a soil auger (1.0 m × 50 mm) at 20 cm depth. Samples were immediately sealed in polyethylene bags, stored in dry ice containers, and transported to the laboratory for preservation at −4 °C.

### 2.2 Isolation of acephate-degrading Bacteria

Acephate-degrading bacteria were isolated from soil using acephate (99.6% purity) as the sole carbon source following the method described by Zhao et al. (2014), involving enrichment, acclimatization, and purification:

1. Ten grams of soil were vortexed with 50 mL of sterile water in a centrifuge tube. The supernatant was transferred to 100 mL nutrient medium (beef extract 3 g, NaCl 5 g, peptone 10 g, deionized water 1,000 mL; pH 6.8) and incubated at 30 °C with shaking at 150 rpm for 24 h;
2. An inoculum was streaked onto nutrient agar plates supplemented with 30 mg L^−1^ acephate and incubated at 30 °C for 48 h. Colonies were subsequently transferred to plates containing 60 mg L^−1^ and 120 mg L^−1^ acephate;
3. Acclimated colonies were streaked onto inorganic salt agar plates containing 240 mg L^−1^ and 500 mg L^−1^ acephate as the sole carbon source (Na_2_HPO_4_ 6.34 g, KH_2_PO_4_ 1.33 g, (NH_4_)_2_SO_4_ 1 g, MgSO_4_·7H_2_O 0.2 g, FeSO_4_ 0.001 g, CaCl_2_ 0.04 g, agar 15 g, deionized water 1,000 mL; pH 6.8) for 10 days incubated at 30 °C;
4. Colonies were subcultured, and bacterial suspensions were serially diluted (10^−5^ to 10^−9^) with sterile water for single-colony isolation. Purified strains were cryopreserved in 20% glycerol at −60 °C (Koch, 2001).

### 2.3 Physiological characterization and 16S rDNA sequencing of bacterial strains

Following isolation, the bacterial strains were streaked onto solid media and incubated at 30 °C for 24 h. Colony morphology, including shape, edge, and pigmentation, was recorded. Gram staining was performed to determine cell wall type. Physiological tests included the methyl red test (MR), Voges–Proskauer test (VP), gelatin hydrolysis, starch hydrolysis, nitrate reduction, and catalase assays (McDevitt, 2009). For temperature tolerance, overnight cultures were inoculated into nutrient medium (pH 6.8) and incubated at 20 °C, 25 °C, 30 °C, 35 °C, and 40 °C with shaking at 150 rpm. Optical density at 600 nm (OD_600_) was measured after 12 h. For pH response, the pH of the nutrient medium was adjusted to 5.0, 6.0, 7.0, 8.0, and 9.0. Inoculated cultures were incubated at 30 °C and 150 rpm, and OD_600_ values were recorded every 4 h intervals.

Genomic DNA was extracted for 16S rDNA sequencing. PCR amplification was performed using primers B341F (5′-CCTACGGGNGGCWGCAG-3′) and B785R (5′-GACTACHVGGGTATCTAAT-3′). The thermal cycling protocol comprised: initial denaturation at 95 °C for 3 min; 25 cycles of denaturation (95 °C, 30 s), annealing (54 °C, 30 s), and extension (72 °C, 30 s); followed by final extension at 72 °C for 5 min. PCR products were verified through 2% agarose gel electrophoresis and submitted to Sangon Biotech Co., Ltd. (Jiangsu, China) for bidirectional sequencing. Sequences were analyzed via the BLAST algorithm against the NCBI database for homology analysis, and phylogenetic trees were reconstructed using MEGA11 software.

### 2.4 Measurement of acephate degradation efficiency

Inorganic salt media containing acephate concentrations of 10, 20, and 50 mg L^−1^ were prepared in 150 mL Erlenmeyer flasks. Each flask was inoculated with bacterial suspension at a 4% inoculum. A sterile filter membrane was secured on flask necks to enable aeration and prevent contamination. Sterile water served as blank controls. Each treatment included three replicates. Flasks were incubated at 30 °C and 150 rpm for 7 days. After incubation, 5 mL aliquots were centrifuged at 4,000 rpm for 3 min. One milliliter of supernatant was filtered through a 0.22 μm membrane, extracted with 10 mL acetonitrile, shaken for 2 min, and sonicated for 20 min. After adding 1 g anhydrous NaCl, the mixture stood for 30 min for phase separation (St-Amand and Girard, 2004). The organic phase was collected for acephate quantification. An additional experiment supplemented with 0.1 g L^−1^ glucose evaluated external carbon source effects on degradation efficiency.

### 2.5 Determination of acephate concentration

Acephate concentration was quantified using gas chromatography–mass spectrometry (GC–MS, GCMS-TQ8040, Shimadzu) equipped with a polar SH-Rxi-17Sil MS column (30 m × 0.25 mm × 0.25 μm). Analytical parameters were as follows: injector temperature 250 °C, interface temperature 280 °C, ion source temperature 240 °C, carrier gas helium (99.999%) at 1.97 mL min^−1^ flow rate, injection volume 1 μL, and selective ion monitoring (SIM) mode. The temperature program was: 65 °C (1 min hold), ramp to 130 °C at 20 °C min^−1^, then to 280 °C at 10 °C min^−1^ (10 min hold), and finally to 300 °C at 10 °C min^−1^ (10 min hold) (Alder et al., 2006).

The standard curve was established by plotting acephate concentration (*x*-axis) against corresponding peak area (*y*-axis), yielding a linear regression equation of *y* = 670,146*x* − 11,6701. The curve exhibited excellent linearity across the concentration range of 0.1, 0.5, 2, 5, and 10 mg L^−1^, as confirmed by a correlation coefficient (*R*^2^) of 0.9995. Method sensitivity was characterized by detection of 0.01 mg L^−1^ (liquid matrices) and 0.6 μg kg^−1^ (soil samples). Validation in an inorganic salt medium spiked with acephate demonstrated acceptable recoveries (93.49–98.73%) and relative standard deviations of 2.47–5.18%, meeting established criteria for pesticide residue analysis.

### 2.6 Combined plant–microbe degradation of acephate

The experimental soil was prepared by blending peat and riparian zone soil (1:1 v/v ratio). The mixture was sterilized by autoclaving and filled into plastic pots (9.8 cm height × 9.8 cm diameter; 300 g/pot), with soil moisture adjusted to 35% water-holding capacity. Two microbial–plant combinations were tested: *Bacillus badius* (DA-3) was paired with its host *Persicaria hydropiper* (30–40 cm height), and *S. spiritivorum* (DA-4) with its host *C. dimorpholepis* (10–15 cm height). Bacterial strains were activated and propagated in nutrient broth. Acephate concentrations in soil were set at 200 μg kg^−1^ and 1,000 μg kg^−1^. Four treatment groups were established: microbial mono-treatment, plant mono-treatment, plant–microbe combination, and blank control (CK) – all with quintuplicate replicates. Incubation proceeded for 30 d in a climate-controlled growth chamber (30 °C). Bacterial inoculum (10 mL) was supplemented weekly, and deionized water was added triweekly to maintain soil moisture.

**Figure 2:**
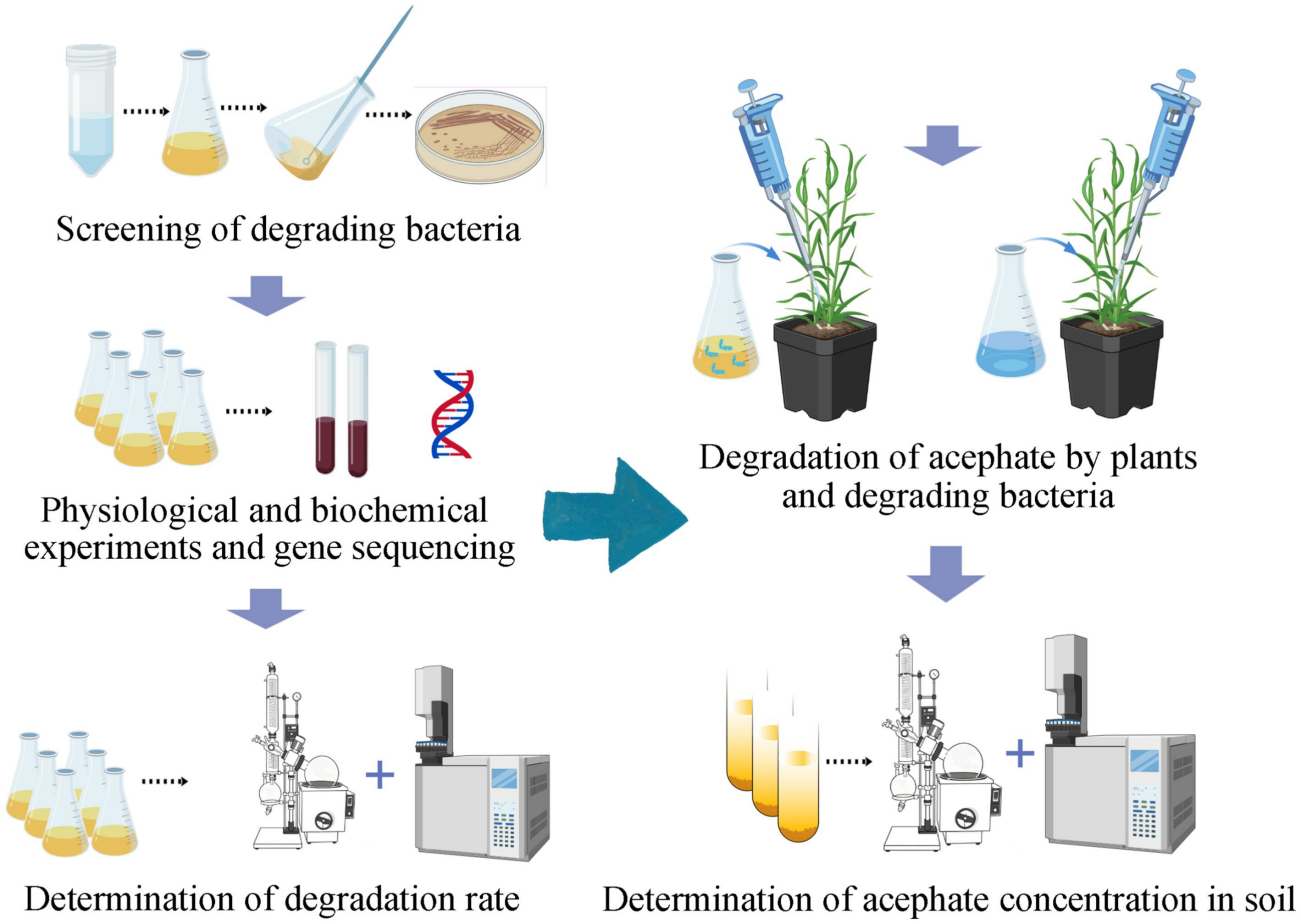
Schematic diagram of the experimental design.

After 30 days, soil samples were collected, freeze-dried, and sieved through a 0.25 mm mesh. Five grams of soil were extracted with 40 mL acetonitrile in 100 mL centrifuge tubes, shaken vigorously, and mixed with 5 g anhydrous NaCl. After centrifugation at 8,000 rpm for 5 min, 10 mL supernatant was transferred to a 100 mL pear-shaped flask and concentrated to ~1 mL a 40 °C. The residue was reconstituted in 3 mL acetonitrile–toluene (1:1), loaded onto a solid-phase extraction (SPE) cartridge, and rinsed twice with 2 mL acetonitrile–toluene. The cartridge was eluted with 25 mL acetonitrile–toluene, and eluents were combined, concentrated to dryness at 40 °C, redissolved in 1 mL ethyl acetate, filtered through an organic microporous membrane, and analyzed via GC–MS. The experimental workflow is depicted in Figure 2.

### 2.7 Data analysis

All data were compiled and preprocessed in Microsoft Excel 2016. Two-way analysis of variance (ANOVA) was performed using SPSS 24.0. When significant differences were identified (*p* < 0.05), Tukey’s HSD test was employed for *post hoc* comparisons. Figures were generated using Python 3.8.

## 3 Results

### 3.1 Morphology and physiological characteristics of acephate-degrading strains

**Figure 3:**
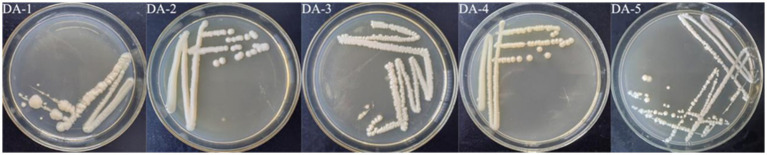
Morphology of the strains on agar medium (DA-1 to DA-5).

Through enrichment, acclimatization, and purification, five target strains were isolated from soil (Figure 3) and designated DA-1, DA-2, DA-3, DA-4, and DA-5, respectively. Gram staining confirmed that DA-3 was Gram-positive, whereas the other four strains were Gram-negative, with all five strains exhibiting entire colony margins (Table 1). Physiological and biochemical characterization are presented in Table 1. MR tests showed DA-4 and DA-5 negative, and the other three positive; VP tests showed DA-4 and DA-5 positive and the other three negative; all strains were gelatin hydrolysis-positive; starch hydrolysis was positive for DA-3 and negative for others; all were nitrate reduction-positive; and all were oxidase-positive.

**Table 1:** Physiological and biochemical identification and morphological description.

Strain	Physiological and biochemical tests							
	MR	VP	Gelatin hydrolysis	Starch hydrolysis	Nitrate reduction	Catalase	Gram stain	Morphology
DA-1	+	−	+	−	+	+	−	Pale gray, smooth edge
DA-2	+	−	+	−	+	+	−	Creamy white, smooth edge
DA-3	+	−	+	+	+	+	+	White, smooth edge
DA-4	−	+	+	−	+	+	−	Yellow, smooth edge
DA-5	−	+	+	−	+	+	−	Creamy yellow, smooth edge

**Figure 4:**
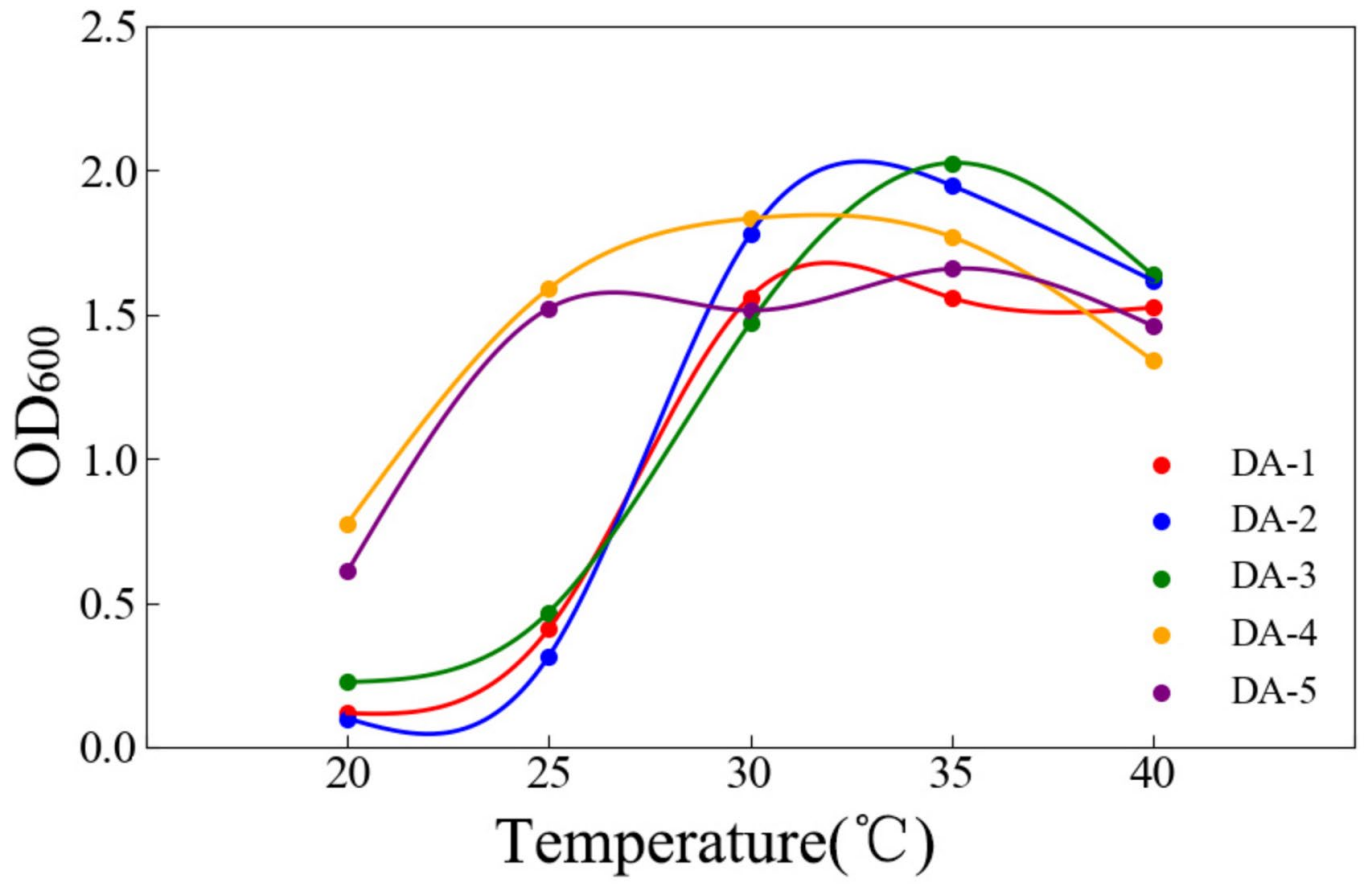
Effects of different temperatures on the growth of degrading strains.

**Figure 5:**
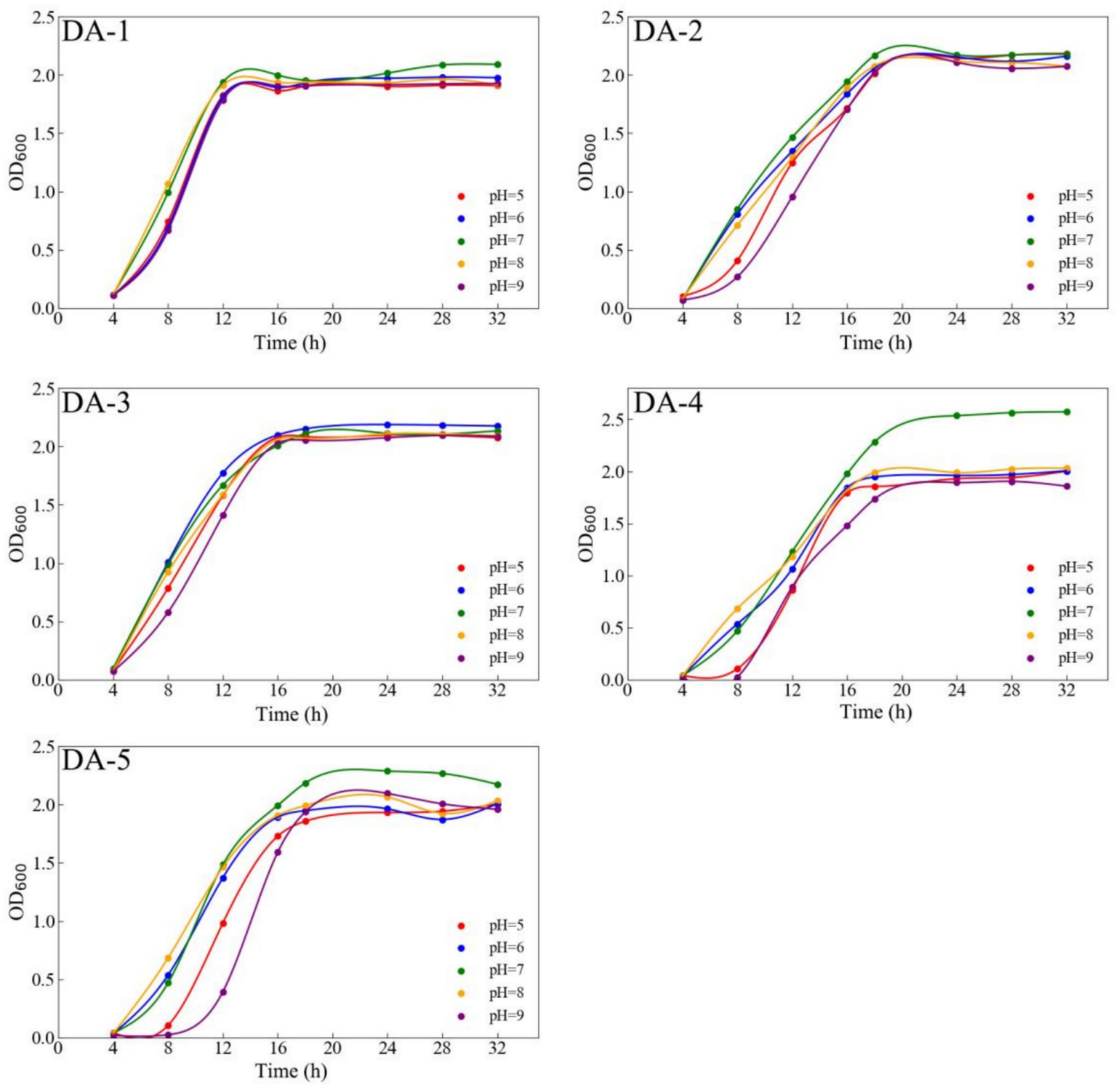
Growth status of acephate-degrading bacteria at different pH.

Temperature significantly influenced the growth of all five strains. Within 20–40 °C, growth initially increased before stabilizing or declining; optimum temperatures for DA-1 through DA-5 were 30, 35, 35, 30, and 35 °C, respectively (Figure 4). pH variations substantially affected DA-4 and DA-5 growth, with significantly higher OD_600_ values at pH 7 versus other pH levels. All strains entered the stationary phase after 16–20 h, with optimal pH values for DA-1 to DA-5 being 7, 7, 6, 7, and 7, respectively (Figure 5).

### 3.2 Molecular identification of the degrading strains

**Figure 6:**
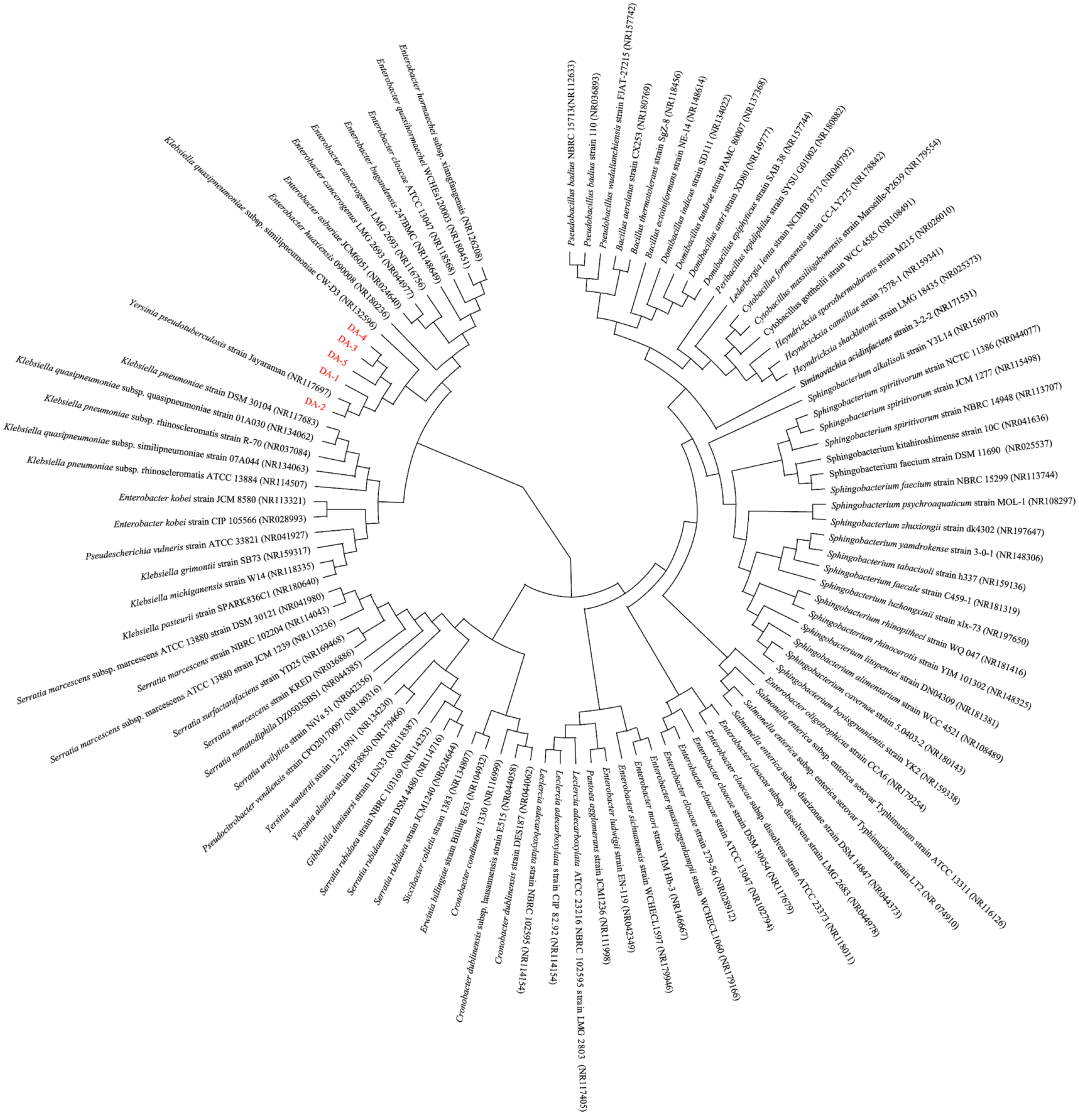
Phylogenetic tree based on 16S rDNA sequences. (DA-1, DA-2, DA-3, DA-4, and DA-5 are the acephate-degrading strains isolated in this study; other microorganisms shown are those known to be most closely related to acephate-degrading bacteria in the NCBI database. The phylogenetic tree was constructed using the neighbor-joining method).

The 16S rDNA sequences of the five strains were analyzed via the BLAST algorithm against the NCBI GenBank database. Combined with their physiological and biochemical characteristics, strains DA-1 and DA-2 were identified as *Enterobacter cloacae* and *E. hormaechei*, respectively. While DA-2 showed the closest phylogenetic proximity to *Pseudotuberculosis,* it was not classified as such. Strain DA-3 was identified as *B. badius* and strain DA-4 as *S. spiritivorum* (with DA-3 and DA-4 being phylogenetically affiliated). Strain DA-5 was identified as *Serratia nematodiphila* (Figure 6). DA-3 belongs to the phylum Firmicutes, DA-4 to Bacteroidota, and the other three strains to Proteobacteria (class *γ*-Proteobacteria, order Enterobacterales, family Enterobacteriaceae). All five strains share close phylogenetic relatedness, with the highest 16S rDNA sequence similarity to *Klebsiella quasipneumoniae.*

### 3.3 Degradation efficiency of acephate-degrading strains

**Figure 7:**
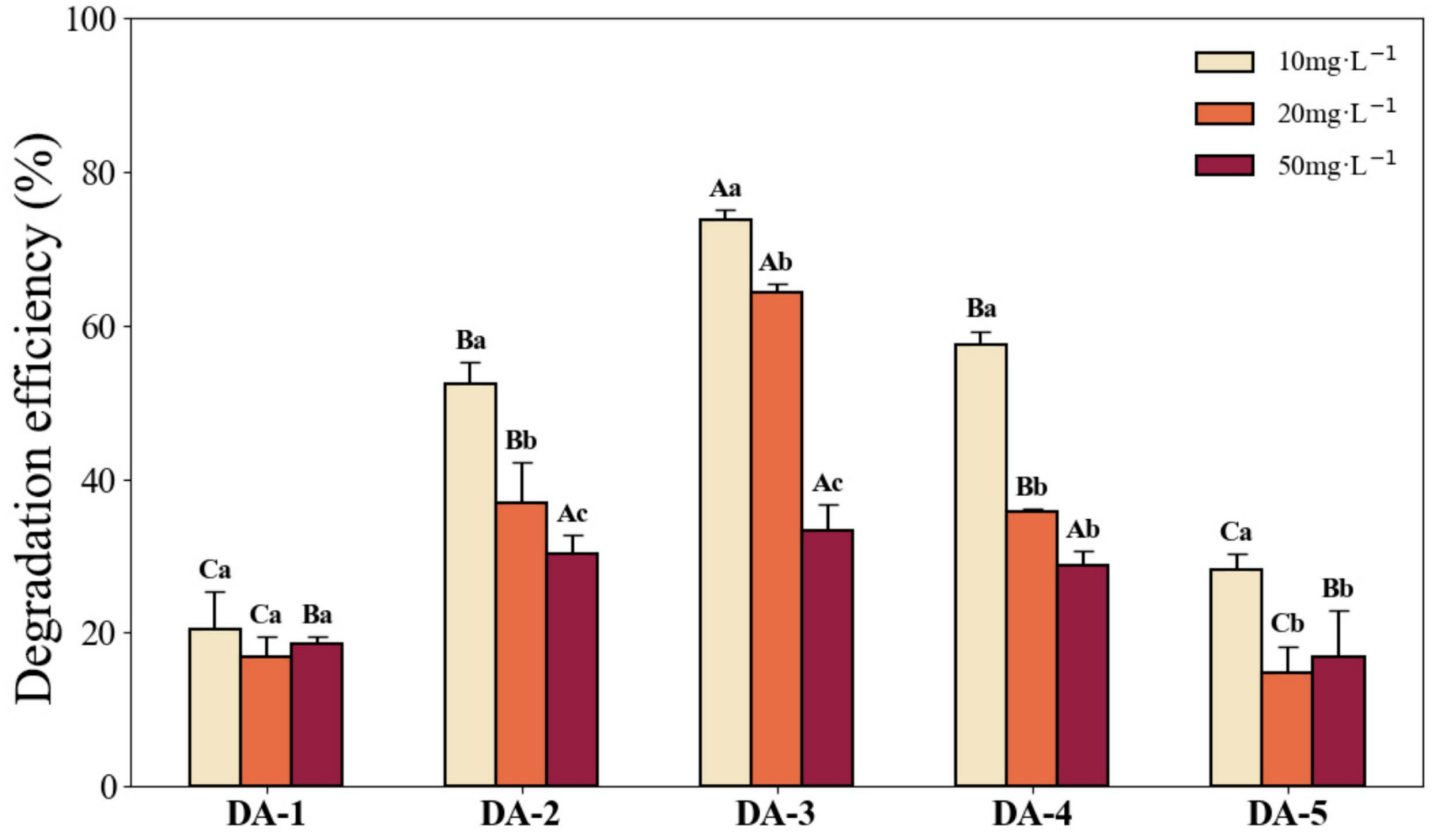
Degradation efficiency of each strain under different acephate concentrations within 7 days. (Different capital letters indicate significant differences in degradation performance among strains at the same acephate concentration; different lowercase letters indicate significant differences in degradation by the same strain at different concentrations. Differences were considered significant at *p* < 0.05).

The acephate degradation capabilities of the five strains are shown in Figure 7. All strains exhibited measurable acephate degradation ability, though efficiencies varied significantly. The degradation rates decreased with increasing acephate concentration. At 10 mg L^−1^, DA-2, DA-3, and DA-4 exceeded 50% degradation, with DA-3 achieving the highest rate at 73.69%, which was significantly higher than the other four strains. At 20 mg L^−1^, DA-3 again exhibited the highest degradation rate (64.36%), significantly higher than the other strains. At 50 mg L^−1^, DA-3 maintained the highest degradation rate (33.24%), being significantly greater than DA-1 and DA-5 (*p* < 0.05) but not significantly different from DA-2 and DA-4.

**Figure 8:**
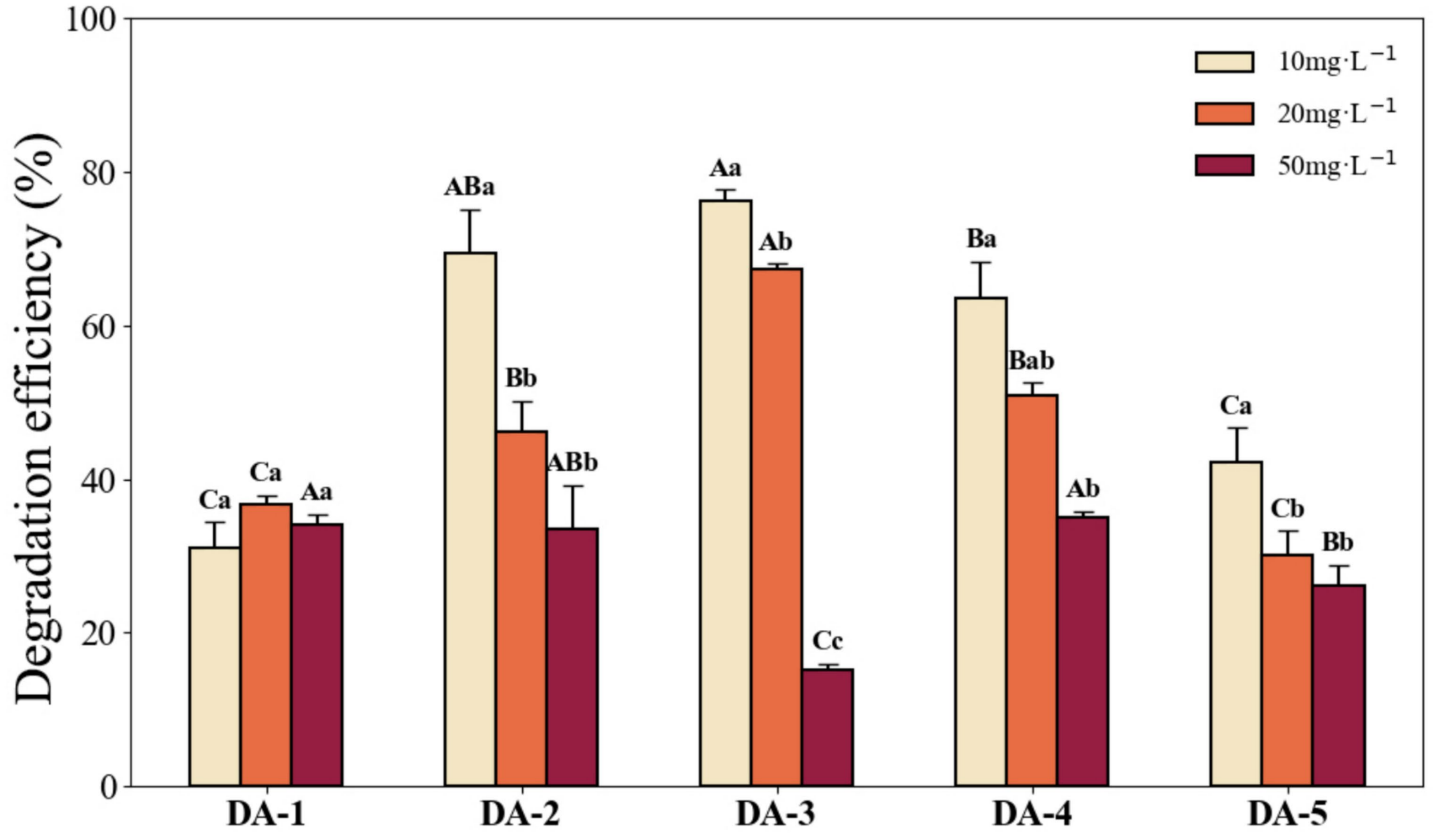
Degradation efficiency of each strain after glucose addition under different acephate concentrations within 7 days. (Different capital letters indicate significant differences among strains at the same acephate concentration; different lowercase letters indicate significant differences within the same strain across different concentrations. Differences were considered significant at *p* < 0.05).

Following supplementation of the inorganic salt medium with 0.1 g L^−1^ glucose, strain degradation efficiencies improved overall (Figure 8), particularly at high acephate concentrations—except for DA-3 at 50 mg L^−1^, which showed no enhancement. The most significant improvement occurred in DA-2 at 10 mg L^−1^, with the degradation rate increasing to 69.47% (a 17.13% increase). At 10 mg L^−1^ acephate, DA-3 maintained the highest degradation rate at 76.17%, significantly higher than DA-1, DA-4, and DA-5, but not significantly different from DA-2 (69.47%). At 20 mg L^−1^, DA-3 achieved the highest degradation rate of 67.22%, significantly surpassing the other strains. At 50 mg L^−1^, DA-4 showed peak degradation (34.97%), significantly higher than DA-3 and DA-5 (*p* < 0.05), but not significantly different from DA-1 and DA-2.

### 3.4 Combined degradation of acephate by plants and degrading strains

**Figure 9:**
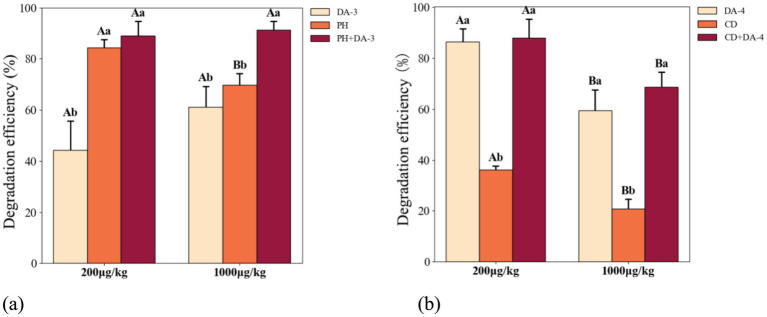
Combined degradation of acephate by different plants and degrading strains. **(a)** DA-3 and Persicaria hydropiper. **(b)** DA-4 and Carex dimorpholepis. (PH: *P. hydropiper*; CD: *C. dimorpholepis*. Different capital letters indicate significant differences between treatments under the same acephate concentration. Different lowercase letters indicate significant differences among treatments at the same concentration. Differences were considered significant at *p* < 0.05).

Plant-microbe combined treatments demonstrated significantly higher acephate degradation efficiency than mono-treatments across concentrations after 30 days (Figure 8). At 200 μg kg^−1^ acephate, the *P. hydropiper*-DA-3 combination achieved a degradation efficiency of 89.15%, which was significantly higher than that of DA-3 alone (44.20%) but not significantly different from that of *P. hydropiper* alone (84.45%, Figure 9a). At 1000 μg kg^−1^, the combination showed peak degradation efficiency (91.27%), significantly higher than both DA-3 (61.11%) and *P. hydropiper* (69.85%).

For the *C. dimorpholepis-*DA-4 combination, the degradation efficiency at 200 μg kg^−1^ reached 87.97%, significantly higher than that of *C. dimorpholepis* alone (36.10%) but not significantly different from that of DA-4 alone (86.40%) (Figure 9b). At 1,000 μg kg^−1^, the combined degradation efficiency was 68.74%, significantly higher than that of both *C. dimorpholepis* alone (20.78%) and DA-4 alone (59.38%). The *P. hydropiper*-DA-3 combination showed no significant difference in degradation between 200 μg kg^−1^ (89.15%) and 1,000 μg kg^−1^ (91.27%) (Figure 9a). Conversely, the *C. dimorpholepis*-DA-4 combination exhibited significantly higher degradation at 200 μg kg^−1^ (87.97%) than at 1,000 μg kg^−1^ (68.74%) (*p* < 0.05; Figure 9B).

## 4 Discussion

### 4.1 Acephate-degrading microorganisms in riparian zones and their degradation capacity

In this study, five acephate-degrading strains were isolated from riparian zone soils along the YC-project. Among them, DA-4 (*S. spiritivorum*) is an oligotrophic bacterium, while the other four strains are eutrophic microorganisms (Koch, 2001). Previous studies have suggested that the nutritional type of microorganisms may influence their biodegradation capacity (Sun et al., 2015). Reported high-efficiency acephate-degrading bacteria are mostly isolated from contaminated sites or industrial effluents. For instance, Ramya et al. (2016) found that *Enterobacter aerogenes* could degrade acephate, which belongs to the same genus as *E. cloacae* and *E. hormaechei* identified in our study. Mohan and Naveena (2015) discovered that *Lysinibacillus* could degrade acephate concentrations up to 500 mg L^−1^. Lin et al. (2016) reported enhanced acephate degradation by *Bacillus subtilis* under Pb^2+^ stress, which is consistent with the genus of *B. badius* (DA-3) in our study. Dong et al. (2024) found that *Serratia marcescens* could degrade acetochlor, which belongs to the same genus as *S. nematodiphila* (DA-5) isolated in our study. Other studies also reported acephate degradation by *Pseudomonas aeruginosa* (Ramu and Seetharaman, 2014) and *Burkholderia* sp. (Wu et al., 2023), which were not observed in this study. This indicates that diverse microbial species can degrade acephate, and the types of microorganisms capable of its degradation vary across ecosystems.

We further assessed the degradation performance of the five isolated strains and found that DA-3 exhibited significantly higher degradation efficiency than the other four strains (Figure 7). This may be attributed to interspecies differences in microbial degradation capacity (Bose et al., 2021). For example, Singh et al. (2020) compared the degradation of acephate by *P. aeruginosa*, *Pseudomonas putida*, and *Pseudomonas azotoformans* under identical conditions, finding *P. aeruginosa* had the highest efficiency, suggesting substantial inter-species variability, consistent with our observations for *E. cloacae* and *E. hormaechei*. Lin et al. (2022) reported that a microbial community degraded acephate most efficiently under conditions of 34.1 °C and pH 8.9, achieving 89.5% at an acephate concentration of 200 mg L^−1^. The lower degradation rate observed in our study may result from differences in microbial species. Numerous studies show that under pure culture conditions, microbial degradation efficiency of organophosphorus pesticides can exceed 90%. For example, *Micromonospora* sp., *Pseudomonas* sp., and *Enterobacter* sp. achieve over 95% degradation or complete mineralization of acephate or methamidophos (Wang et al., 2010; Li et al., 2014; Singh et al., 2017). In this study, *B. badius* (DA-3) showed a maximum degradation rate of 76.17%, which is potentially due to the influence of culture time or pesticide type (Guerrero Ramírez et al., 2023). However, our findings align with Phugare et al. (2012), who found that *Exiguobacterium* sp. degraded acephate with a rate of 75.85%, possibly reflecting shared Bacillaceae phylogeny. These findings further support our speculation that variations in acephate degradation efficiency are due to strain-specific characteristics.

We also observed reduced microbial degradation efficiency with increasing acephate concentration, mitigated by glucose supplementation, especially at high acephate concentrations. This may be due to the stimulatory effect of additional carbon sources on microbial growth (Li et al., 2021), which enhanced degradation activity. This suggests that nutrient stress limits acephate biodegradation.

### 4.2 Combined plant–microbe remediation of acephate

Under natural conditions, plants secrete extracellular enzymes such as esterases and phosphatases through their roots to promote pesticide degradation. Esterases hydrolyze ester bonds, while phosphatases cleave phosphate ester bonds in organophosphorus pesticides, reducing pesticide concentrations (Sun et al., 2010). However, plant root degradation of organophosphorus compounds is often slow and subject to low tolerance (Li and Fantke, 2023). Microorganisms, although efficient degraders, are often limited by environmental factors and nutrient availability. In nutrient-deficient soils, microbial activity may be severely inhibited (Zhou et al., 2020). Plants can offset this limitation by releasing carbon and nitrogen sources through fine root turnover and root exudates, thus promoting microbial growth (Yu et al., 2022). Therefore, plant–microbe combined remediation has emerged as an effective strategy for enhancing pesticide degradation. For example, Vaishnavi and Osborne (2024) reported that co-remediation using *Proteus myxofaciens* and *Chrysopogon zizanioides* achieved over 90% degradation of monocrotophos after 45 days. Jabeen et al. (2016) found that combining *Lolium perenne* with endophytic rhizobia promoted bacterial rhizosphere colonization, aiding chlorpyrifos removal. Our results demonstrated that combining strains with the plant under different concentrations enhanced acephate degradation, consistent with these studies (Xie et al., 2018). Consequently, our findings confirm that plant–microbe interactions accelerate acephate degradation in contaminated soils.

Notably, under 200 μg kg^−1^ acephate, the *P. hydropiper* and DA-3 combination achieved 89.15% degradation. When acephate concentration increased to 1,000 μg kg^−1^, the degradation rate rose to 91.27% (though statistically insignificant, *p* > 0.05). This unexpected result may stem from DA-3 increasing its degradation from 44.20 to 61.11% under higher concentrations. We speculate that elevated pesticide levels induced DA-3 to secrete broad-spectrum enzymes (phosphatases and sulfur oxidases), enabling co-metabolism of acephate and root-derived carbon sources, thereby mitigating toxicity stress (Ke et al., 2023). For instance, Yu et al. (2010) reported that their acephate-degrading strain Y1 exhibited increased degradation at 100–500 mg L^−1^. Furthermore, the *P. hydropiper*-DA-3 combination at 1,000 μg kg^−1^ (91.27%) outperformed the *C. dimorpholepis*-DA-4 combination. This may be attributed to the extensive rhizosphere of *P. hydropiper* in moist soils, forming dense root networks that enhance microbial colonization. Previous studies indicate that flavonoids in *P. hydropiper* root exudates induce oph gene expression in Enterobacter, facilitating cleavage of P–O alkyl bonds in organophosphates (Li et al., 2020). We thus speculated that these exudates potentially promoted *B. badius* enrichment and *oph* gene expression, contributing to the high degradation. In contrast, phenolic compounds such as ferulic acid secreted by *C. dimorpholepis* may inhibit enzyme activity in *Sphingobacterium* sp. (Güsewell and Schroth, 2017). Moreover, *P. hydropiper* is a dicotyledonous plant, while *C. dimorpholepis* is monocotyledonous, and differences in plant morphology and physiology may also influence degradation capacity (Sreenivasulu and Wobus, 2013; Wang et al., 2023).

Numerous studies demonstrate that hydrolysis, dehalogenation, and oxidation are enzymatic reactions driving organophosphorus pesticide degradation (Bosu et al., 2024; Bosu et al., 2024). Consequently, this degradation proceeds through distributed pathways, yielding diverse intermediate metabolites and terminal products. For example, Kumari et al. (2025) identified acetamide and trimethyl phosphate as intermediate metabolites of monocrotophos. Hou et al. (2021) reported that dialkyl phosphates are the predominant terminal metabolites in bacterial degradation of nonhalogenated organophosphate esters. Sur and Sathiavelu (2024) identified methyl diethanolamine and aspartyl glycine ethyl ester as intermediate metabolites in dimethoate degradation, with O, O, S-trimethyl phosphorothioate as the terminal metabolite. Consequently, terminal metabolites of organophosphorus pesticides are typically non-toxic phosphate derivatives (Das et al., 2024). Therefore, although degradation products of acephate were undetected in this study, we infer its mineralization to non-toxic phosphate, thereby achieving the purpose of pollution remediation. In summary, this work lies not only in the first-time isolation of an acephate-degrading microbe from the riparian zones but also in demonstrating its synergistic potential with plants—achieving over 90% degradation and thus opening avenues for *in situ* bioremediation.

## 5 Conclusion

In this study, five acephate-degrading microbial strains were isolated from riparian zone soils along the project of water diversion from the Yangtze River to Chaohu Lake. These strains were identified as *E. cloacae*, *E. hormaechei*, *B. badius*, *S. spiritivorum*, and *S. nematodiphila*. Culture experiments demonstrated that *B. badius* exhibited the highest degradation efficiency for acephate. Increasing acephate concentrations generally reduced microbial degradation efficiency, whereas the addition of glucose alleviated this inhibitory effect (except for *B. badius* at 50 mg L^−1^ acephate). Combined degradation experiments showed that at 1,000 μg kg^−1^ acephate, the co-remediation by *B. badius* and *P. hydropiper* achieved optimal degradation, with a removal rate of 91.27%. These findings provide a scientific basis for the bioremediation of acephate-contaminated soils.

## Data Availability

The raw data supporting the conclusions of this article will be made available by the authors, without undue reservation.
